# Novel fusion protein PK5-RL-Gal-3C inhibits hepatocellular carcinoma via anti-angiogenesis and cytotoxicity

**DOI:** 10.1186/s12885-023-10608-9

**Published:** 2023-02-15

**Authors:** Xiaoge Gao, Pin Jiang, Xiaohuan Wei, Wei Zhang, Jiwei Zheng, Shishuo Sun, Hong Yao, Xiangye Liu, Qing Zhang

**Affiliations:** 1 Cancer Institute, Xuzhou Medical University, Xuzhou, Jiangsu Province 221004 People’s Republic of China; 2 Center of Clinical Oncology, The Affiliated Hospital of Xuzhou Medical University, Xuzhou, Jiangsu Province 221004 People’s Republic of China; 3 Jiangsu Center for the Collaboration and Innovation of Cancer Biotherapy, Xuzhou Medical University, Xuzhou, Jiangsu Province 221004 People’s Republic of China; 4 Nanjing International Hospital Co., Ltd., Nanjing, Jiangsu Province 210000 People’s Republic of China; 5 Medical Oncology of Huangmei People’s Hospital, Huanggang, Hubei Province 435500 People’s Republic of China; 6 Department of Oral Medicine, School of Stomatology, Xuzhou Medical University, Xuzhou, Jiangsu 221004 People’s Republic of China; 7 Department of Cancer Biotherapy Center, Third Affiliated Hospital of Kunming Medical University, Kunming, Yunnan Province 650118 People’s Republic of China; 8 Department of Pathogenic Biology and Immunology, Jiangsu Key Laboratory of Immunity and Metabolism, Xuzhou Medical University, Xuzhou, Jiangsu Province 221004 People’s Republic of China

**Keywords:** Galectin-3, Angiogenesis, Carbohydrate-recognition domain, Fifth kringle of plasminogen, Hepatocellular carcinoma

## Abstract

**Background:**

Galectin-3 (Gal-3), the only chimeric β-galactosides-binding lectin, consists of Gal-3N (N-terminal regulatory peptide) and Gal-3C (C-terminal carbohydrate-recognition domain). Interestingly, Gal-3C could specifically inhibit endogenous full-length Gal-3 to exhibit anti-tumor activity. Here, we aimed to further improve the anti-tumor activity of Gal-3C via developing novel fusion proteins.

**Methods:**

PK5 (the fifth kringle domain of plasminogen) was introduced to the N-terminus of Gal-3C via rigid linker (RL) to generate novel fusion protein PK5-RL-Gal-3C. Then, we investigated the anti-tumor activity of PK5-RL-Gal-3C in vivo and in vitro by using several experiments, and figured out their molecular mechanisms in anti-angiogenesis and cytotoxicity to hepatocellular carcinoma (HCC).

**Results:**

Our results show that PK5-RL-Gal-3C can inhibit HCC both in vivo and in vitro without obvious toxicity, and also significantly prolong the survival time of tumor-bearing mice. Mechanically, we find that PK5-RL-Gal-3C inhibits angiogenesis and show cytotoxicity to HCC. In detail, HUVEC-related and matrigel plug assays indicate that PK5-RL-Gal-3C plays an important role in inhibiting angiogenesis by regulating HIF1α/VEGF and Ang-2 both in vivo and in vitro. Moreover, PK5-RL-Gal-3C induces cell cycle arrest at G1 phase and apoptosis with inhibition of Cyclin D1, Cyclin D3, CDK4, and Bcl-2, but activation of p27, p21, caspase-3, -8 and -9.

**Conclusion:**

Novel fusion protein PK5-RL-Gal-3C is potent therapeutic agent by inhibiting tumor angiogenesis in HCC and potential antagonist of Gal-3, which provides new strategy for exploring novel antagonist of Gal-3 and promotes their application in clinical treatment.

**Supplementary Information:**

The online version contains supplementary material available at 10.1186/s12885-023-10608-9.

## Introduction

Angiogenesis is a complicated process of new blood vessels formation from preexisting capillaries that is driven by up-regulated pro-angiogenic factors under tumor microenvironments, *e. g.*, vascular endothelial growth factor (VEGF) [1]. Angiogenesis has become an enticing and critical therapeutic target for various malignancies depending on its close association with tumor growth especially for advanced hepatocellular carcinoma (HCC) that has been characterized by hypervascularization [2, 3]. Interestingly, sorafenib and lenvatinib, as the first-line therapy for advanced HCC, could inhibit tumor angiogenesis by targeting multiple tyrosine kinases, such as VEGF receptor 1–3, platelet-derived growth factor receptor (PDGFR) α and β, RET, as well as KIT [4, 5]. In addition, monoclonal antibodies directly targeting VEGF, PDGF, angiopoietin-2 (Ang-2), and their receptors are also used in the clinical or pre-clinical cancer treatment. Furthermore, some of them have been approved for clinical cancer therapy by Food and Drug Administration of the Unites States [6]. Whereas, most anti-angiogenesis therapy exhibits limited effects because of the development of drug resistance and intrinsically low response. Thus, it is still urgent to explore more effective therapy targets and antagonists of tumor angiogenesis.

Galectin-3 (Gal-3), a β-galactosides-binding lectin, is the only chimeric member of galectins family, which consists of two functional domains including unique N-terminal regulatory domain (Gal-3N) and C-terminal carbohydrate-recognition domain (Gal-3C) [7]. Increasing evidence has demonstrated that Gal-3 is highly expressed in many cancer cells and secreted via non-classical secretary pathway, which contributes to tumor progression and metastasis [7, 8]. Previous studies have demonstrated that Gal-3 is considered as a potential target for anti-angiogenesis therapy [9].

Interestingly, several investigations have proved that Gal-3C could inhibit the function of full-length galectin-3 via competitively binding with its endogenous ligands [10–12]. Thus, Gal-3C acts as a potent inhibitor of full-length Gal-3. Importantly, Gal-3C treatment showed low toxicity and well-tolerated that is significant for its potential utility as antineoplastic drugs. Taken together, Gal-3C might be a specific competitive motif to inhibit endogenous full-length Gal-3, which drove us to suppose that further modification of Gal-3C could generate novel fusion proteins with better anti-tumor activity. PK5, the fifth kringle domain of plasminogen, comprises of 80 amino acids and can form triple loop structure by 3 disulfide linkages [13]. Previous evidences showed that PK5 could effectively inhibit angiogenesis both in vitro and in vivo and also exhibit anti-tumor activity in HCC [14], gastric cancer [15], and lung cancer [16]. Therefore, PK5 is a potential antineoplastic reagent.

Considering high efficacy, small molecular weight, and stable structure of PK5 as well as specific inhibiting endogenous Gal-3 of Gal-3C, we firstly constructed novel fusion protein PK5-RL-Gal-3C via linking PK5 and Gal-3C domains with rigid linker (RL). Our results revealed that the novel chimeric protein PK5-RL-Gal-3C exhibited stronger anti-tumor activity and significantly prolonged the survival time of tumor-bearing mice in orthotopic mouse liver cancer model compared to PK5 or Gal-3C treatment alone. Furthermore, PK5-RL-Gal-3C suppressed angiogenesis both in vitro and in vivo in Gal-3C-dependent and -independent manners via regulating the expression of HIF1α/VEGF and Ang-2. Surprisingly, the fusion protein could also inhibit HCC cell proliferation in Gal-3C dependent manners by inducing cell cycle arrest and apoptosis through reversing the expression changes of cell cycle and apoptosis associated proteins. In conclusion, the successful construction of novel fusion protein PK5-RL-Gal-3C based on Gal-3C provides theoretical basis for their further clinical application in the treatment of HCC patients and offers new strategy for further exploring Gal-3 antagonist.

## Materials and methods

### Cell lines and antibodies

HCC cell lines including HepG2, Huh7, and PLC/PRF/5 as well as mouse liver cancer cell line ML-1 were all cultured in Dulbecco’s modified eagle medium supplemented with 10% heat-inactivated fetal bovine serum and 1% penicillin and streptomycin. All the HCC cell lines were purchased from the national collection of authenticated cell cultures (Shanghai, China) and have been authenticated using STR (or SNP) profiling within the last three years. Mouse liver cancer cell line ML-1 was kindly provided by Dr Che-Hsin Lee [17]. Human umbilical vein endothelial cells (HUVECs) were purchased from Procell Life Science and Technology (Wuhan, China). All the cultures were incubated at 37°C in a humidified incubator with 5% CO_2_. The antibodies of GAPDH (5174), caspase-8 (9746), caspase-9 (9504), caspase-3 (9664), PARP (9532), Bcl-2 (4223), Bax (2772), CDK4 (12,790), Cyclin D1 (2798) were all purchased from Cell Signaling Technology (Danvers, MA, USA). The antibody against tubulin (HC101-01) was purchased from TransGen Biotechnology (Beijing, China). The antibodies of CD31 (ab28364) and Ki-67 (ab16667) were purchased from Abcam (Cambridge, MA, USA). The antibodies against p27 (25614–1-AP), p21 (10355–1-AP), Cyclin D3 (26755–1-AP), VEGF-A (19003–1-AP), HIF-1α (20960–1-AP), Ang-2 (11992–1-AP), and CD34 (60180–2-Ig) were purchased from Proteintech Group (Wuhan, China). All the secondary antibodies labeled with HRP were purchased from Beyotime Biotechnology (Shanghai, China). Deferoxamine (DFO) and sorafenib tosylate was purchased from TargetMol (Shanghai, China). Tribromoethyl alcohol was purchased from Merck (Shanghai, China).

### Animals

Female wild type BALB/c mice and C57BL/6 mice (six to eight weeks old) were purchased from Beijing Vital River Laboratory Animal Technology Co., Ltd (Beijing, China) and maintained at the Animal Center of Xuzhou Medical University. Animal study was conducted in strict accordance with the recommendations of the Experimental Animal Care and Use Guidelines of the Experimental Animal Ethics Committee of Xuzhou Medical University. The program was approved by the Experimental Animal Ethics Committee of Xuzhou Medical University (Permit Number: 201547).

### Preparation of eukaryotic expression plasmids

Firstly, the eukaryotic expression vector CAGs-GFP-T2A-Luciferase-Enhanced Episomal Vector (EEV) (SBI system biosciences, Palo Alto, CA, USA) was simultaneously digested by EcoRI and XhoI. The cDNA sequence encoding IgK leader peptide and PK5, Gal-3C, or PK5-RL-Gal-3C as well as ligating EcoRI and XhoI cutting sites were synthesized in GENEWIZ (Suzhou, China). Then, all cDNA fragments were respectively inserted into EEV vector to generate plasmids EEV-PK5, EEV-Gal-3C, and EEV-PK5-RL-Gla-3C. All the plasmids were sequenced and then purified with PureLink™ Hipure plasmid maxiprep kit (Invitrogen, CA, USA) for further used.

### Preparation of H1/plasmid nanoparticles

Firstly, delivery vector H1 was prepared according to the method reported previously [18]. Then, H1 was respectively mixed with plasmids EEV-PK5, EEV-Gal-3C, and EEV-PK5-RL-Gla-3C dissolved in solution buffer (5% sucrose solution) at the indicated N/P ratios (20:1, where ‘N’ is the amount of nitrogen in PEI and ‘P’ is the amount of phosphate in 1 μg of DNA). Then, the H1/plasmid nanoparticles were freeze-dried and re-suspended in deionized water prior to injection.

### Orthotopic mouse liver cancer model

Orthotopic mouse liver cancer model was established with mouse liver cancer cell line ML-1 according to previous description [19]. Briefly, ML-1 cells were collected and injected slowly into the left lobe of liver of anesthetized BALB/c mice with tribromoethyl alcohol. Four days after tumor cell implantation, all mice were randomly grouped and treated by intraperitoneal injection with freeze-dried H1/nanoparticles of EEV, EEV-PK5, EEV-Gal-3C, and EEV-PK5-RL-Gal-3C (100 μg per mouse) once a week, respectively. Sorafenib was given by oral administration at the dosage of 50 mg/kg mouse body weight every two days. After four weeks, tumor-bearing mice from each group were anesthetized by intraperitoneal injection of tribromoethyl alcohol and then killed by cervical dislocation. The tumor body was removed and weighed. Five mice in each group were kept for the survival investigation.

### Preparation of recombinant protein rPK5, rGal-3, rGal-3C, and rPK5-RL-Gal-3C

The cDNA of recombinant protein rPK5, rGal-3C, and rPK5-RL-Gal-3C were respectively constructed into prokaryotic expression vector pET-22b ( +) and expressed in *E. coli* BL 21 (DE3) through induced by IPTG. The rPK5 with 6 × His tag at the C-terminus was purified with His-tag purification resin (Beyotime Biotechnology, Shanghai, China), and rGal-3C and rPK5-RL-Gal-3C were purified with α-lactose-agarose (Sigma-Aldrich, Shanghai, China) according to commercial introductions. After diluted in PBS buffer, the purity and concentration of recombinant proteins were respectively analyzed with SDS-PAGE and BCA protein concentration kit. In addition, the endotoxin levels of all recombinant proteins were measured using ToxinSensor™ endotoxin detection system (GenScript, Nanjing, China).

### Tube formation assay in vitro

Primarily, 100 μl matrigel was used to cover the bottom for each well of 48-well plate, and then the plate was incubated for 30 min at 37 °C for solidification. HUVECs were collected and suspended in EGM without serum, and then mixed with rPK5, rGal-3C, rPK5-RL-Gal-3C, and E25 (N-terminal 25 amino acids of human endostatin, as positive control) under the same final concentration of 2 μmol/l, respectively. Subsequently, the mixture was added into each well coated with matrigel. After 6–8 h incubation in a 37 °C and 5% CO_2_ incubator, the formed tubes were photographed and counted using an inverted microscope (Olympus Life Science, Tokyo, Japan). In the present assay, 5 mg/ml of lactose was set as positive control to specifically inhibit Gal-3C, otherwise the same concentration of sucrose was used as negative control.

### Matrigel plug assay in vivo

Growth factor reduced matrigel without phenol red (Corning, Shanghai, China) was well mixed with 100 ng/ml VEGF-A165 (PeproTech, NJ, USA) on ice. Then, rPK5, rGal-3C, rPK5-RL-Gal-3C were added into the mixture with the final concentration of 2 μmol/l, respectively. Subsequently, the mixture was subcutaneously injected into the flank of C57BL/6 mice. One week after injection, mice were sacrificed and plugs were removed and photographed.

### Cell viability assay

The effects of rPK5, rGal-3C, and rPK5-RL-Gal-3C on HCC cell viability were measured with a MTT assay kit (VICMED, Xuzhou, China). Briefly, tumor cells were planted on 96-well plates. Next day, the medium was replaced with new medium containing purified rPK5, rGal-3C, or rPK5-RL-Gal-3C under the indicated concentrations (0, 1, 2, 3 and 4 μmol/l), and then incubated for further 48 h. Following added MTT reagent and incubated for 4 h, the absorbance was read at 570 nm with a full wavelength microplate reader (BioTek Instruments, VT, USA).

### Cell cycle assay

Following treated with 4 μmol/l of rPK5-RL-Gal-3C for 48 h, HCC cells were fixed with 70% pre-chilled ethanol overnight at 4 °C, and then stained with PI (KeyGen Biotech, Nanjing, China) at 37 °C for 30 min in dark. Finally, the cells were analyzed with BD FACSCanto™ II Cell Analyzer (BD Biosciences, CA, USA).

### Cell apoptosis assay

After treated with 4 μmol/l of rPK5-RL-Gal-3C for 48 h, HCC cells were stained with Annexin V-FITC/PI apoptosis detection kit (KeyGen Biotech, Nanjing, China) according to the manufacturer’s protocol. Then, the cells were analyzed with BD FACSCanto™ II Cell Analyzer.

### SDS-PAGE and Western-blotting assay

After treated with 4 μmol/l of rPK5-RL-Gal-3C for 48 h, HCC cells were harvested and lysed in RIPA buffer with protease inhibitor on ice for 30 min. Then the supernatant was collected after centrifuging at 13, 000 g for 15 min and the protein concentrations were measured by using BCA protein concentration assay kit (Vazyme, Nanjing, China). Subsequently, 30 μg of protein was separated with SDS-PAGE and transferred to the PVDF membrane. After blocked with 5% nonfat-dried milk for 1 h at room temperature, the membranes were cropped according to the related protein molecular weight. Only targeted blots were reserved and incubated with primary antibodies overnight at 4 °C. Following incubated with secondary antibodies for 1 h at room temperature, the immunodetection of the membrane was performed using *Tanon* imaging system (Tanon Science & Technology Co, Ltd, Shanghai, China).

### Immunohistochemistry (IHC) and Hematoxylin–Eosin (HE) Staining

After fixed with 10% neutral formalin buffering solution, the paraffin embedded tumors were cut into sections with 4 μm thickness. Following deparaffinized and rehydrated, the antigen retrieval process of tumor sections was performed in 10 mM sodium citrate buffer (pH 6.0) by using microwave. After blocked endogenous peroxides activity with 3% H_2_O_2_ for 10 min, tumor sections were gently washed with PBS and subsequently incubated with primary antibodies overnight at 4 °C, respectively. Following incubated with secondary antibodies at room temperature for 30 min, tumor sections were stained by DAB. Finally, at least three fields per section were analyzed and positive cells were counted with Image-Pro Plus 7.0 (Media Cybernetics, Inc., MD, USA).

For HE staining, the fixed mouse internal organs including heart, liver, spleen, lung, and kidney were also sectioned into 4 μm and stained using an HE staining kit (Beyotime Technology, Shanghai, China) according to the manufacturer’s protocols. Tissue sections were observed under an upright microscope (Olympus Life Science, Tokyo, Japan), and at least three fields per section were analyzed.

### Statistical assay

In the present study, all data analysis was performed with Graphpad Prism 8.0.1 (GraphPad Software, Inc. CA, USA), and a value of *p* < 0.05 was considered to be significant difference. The in vitro experiments were repeated at least three times and the data was presented as mean ± standard deviation (SD). Two-tailed independent student’s *t* test was performed to analyze two-group comparison assuming equal variances. One-way analysis of variance ANOVA was used to analyze multiple group comparisons following normality and log normality tests and Kaplan–Meier method was used for survival analysis.

## Results

### Preparation of recombinant proteins rPK5, rGal-3C, and rPK5-RL-Gal-3C

**Fig. 1:**
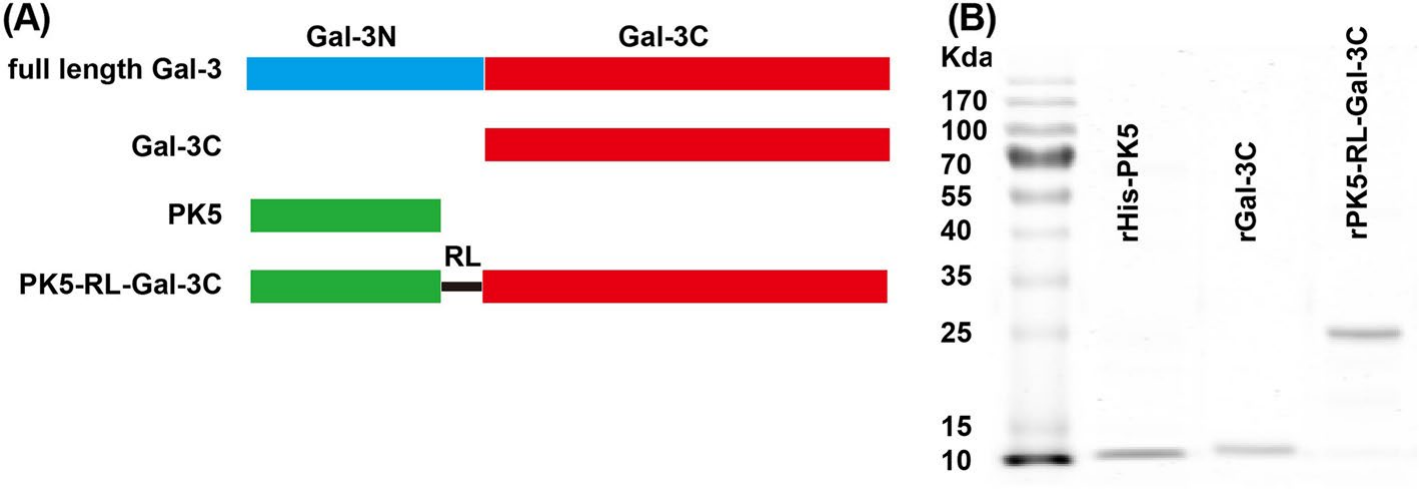
Preparation of recombinant protein PK5, Gal-3C, and PK5-RL-Gal-3C. **A** Schematic structure of full-length Gal-3, PK5, Gal-3C, and PK5-RL-Gal-3C. **B** Purity of recombinant proteins was analyzed by using 15% SDS-PAGE. rPK5 is composed of 80 amino acid residues with 6 × His tag at its N-terminus for purification. rGal-3C contains 107 to 250 amino acid residues of full-length Gal-3. RL is composed of 12 amino acids (AEAAAKEAAAKA)

According to the materials and methods, gene fragments encoding rPK5, rGal-3C, and rPK5-RL-Gal-3C were respectively cloned into prokaryotic expression plasmid pET-22b ( +) (Fig. 1A) and then transfected *E. coli* BL21 (DE3) cells. Following purified with His-tag purification resin for rPK5 tagged 6 × His tag at its C-terminus (88 amino acids), α-lactose agarose for rGal-3C (107 amino acids) and rPK5-RL-Gal-3C (236 amino acids), the purity of all proteins was examined by using SDS-PAGE. The endotoxin levels of all recombinant proteins were low enough for all in vivo and in vitro experiments (Fig. S1). High purity and yield of rPK5-RL-Gal-3C (Fig. 1B) implied that our fusion expression system did not affect the carbohydrate binding activity of Gal-3C domain.

### PK5-RL-Gal-3C inhibits HCC tumor growth in vivo

**Fig. 2:**
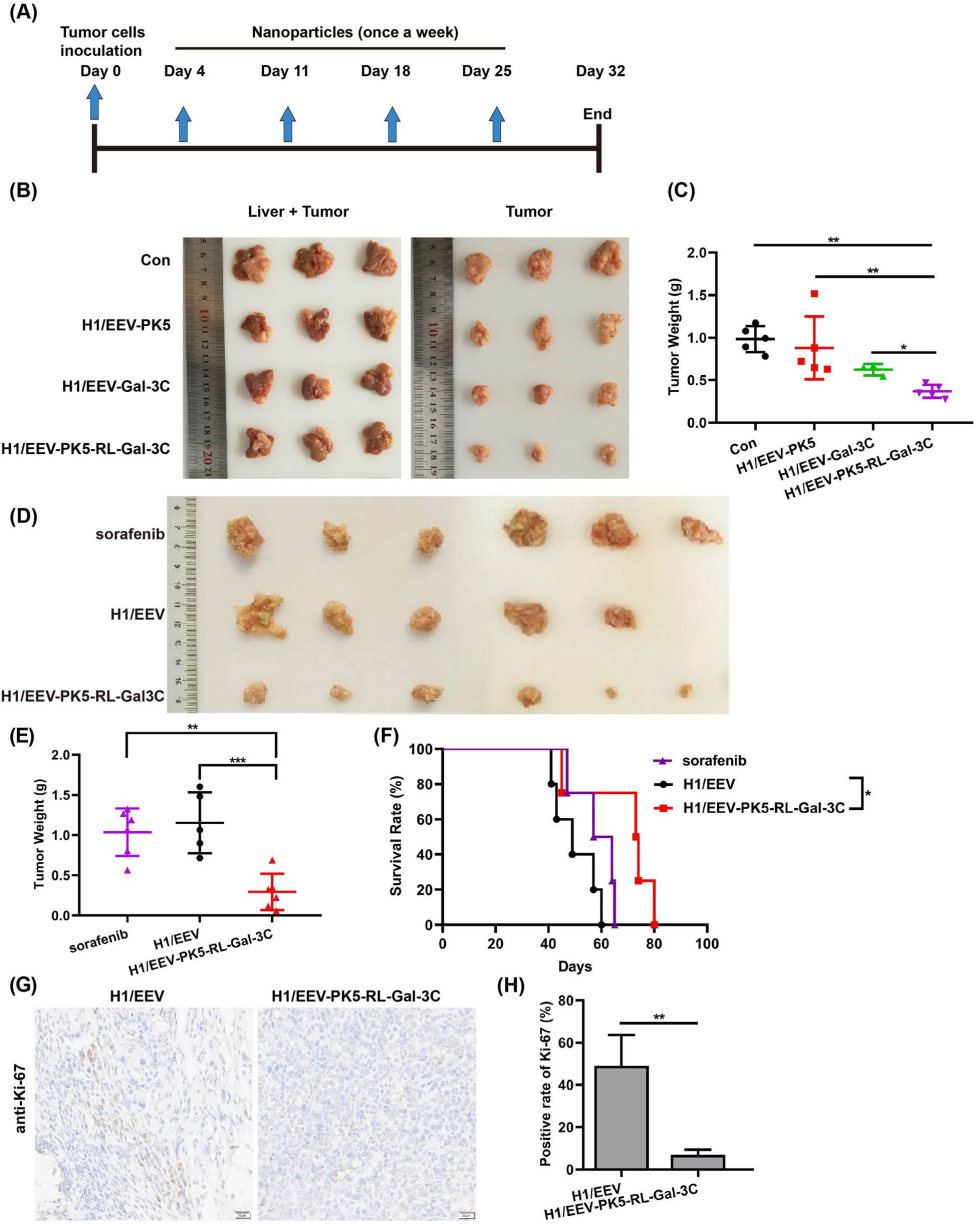
PK5-RL-Gal-3C inhibits tumor growth in orthotopic mouse liver cancer model and prolongs the survival time of tumor-bearing mice. Orthotopic mouse liver cancer model was successfully established with mouse liver cancer cells—ML-1 according to the materials and methods. **A** Schematic diagram illustrated the therapeutic schedule. **B** and **C** H1/EEV-PK5-RL-Gal-3C (*n* = 5) nanoparticles treatment significantly inhibited tumor growth compared to the control (*n* = 5), H1/EEV-PK5 (*n* = 5), and H1/EEV-Gal-3C (*n* = 3). **D** and **E** H1/EEV-PK5-RL-Gal-3C (*n* = 6) nanoparticles treatment inhibited tumor growth compared to the control (H1/EEV, *n* = 5) and sorafenib (*n* = 6) with significant difference. **F** H1/EEV-PK5-RL-Gal-3C (*n* = 4) treatment significantly prolonged the survival time of tumor-bearing mice compared to the control (H1/EEV, *n* = 5). **G** and **H** After treated with H1/EEV and H1/EEV-PK5-RL-Gal-3C nanoparticles, the tumors were removed and stained by Ki-67 antibody using IHC. H1/EEV-PK5-RL-Gal-3C treatment down-regulated the expression of Ki-67 in tumor tissues. Scale bar = 20 μm. Significant differences are denoted by * for *p* < 0.05, and ** for *p* < 0.01

In order to investigate the anti-tumor activity of our fusion protein PK5-RL-Gal-3C in vivo, orthotopic mouse liver cancer models were established according to the description in materials and methods. Moreover, the nanoparticles composed of H1 (gene delivery vector in vivo) and eukaryotic expression plasmid EEV (expressing PK5, Gal-3C, and PK5-RL-Gal-3C with signal peptide IgK, respectively) were prepared according to our previous description [20]. After treated for 4 weeks with the above nanoparticles (Fig. 2A), mice were sacrificed and tumors were removed and weighed. As shown in Fig. 2B and C, all treatment exhibited inhibition on tumor growth compared to the control that treated with deionized water. Interestingly, PK5 and Gal-3C inhibited 26.8% and 36.5% tumor growth, respectively. Surprisingly, PK5-RL-Gal-3C exhibited 62.4% inhibitory rate.

Considering the effect of gene delivery vector H1 to tumor growth, we further set up H1/EEV group as negative control, which just received nanoparticles composed of H1 and empty EEV plasmids. As shown in Fig. 2D and E, PK5-RL-Gal-3C treatment significantly exhibited 74.6% inhibitory rate compared to the negative control. Furthermore, PK5-RL-Gal-3C treatment significantly prolonged the survival time of tumor-bearing mice compared to negative control (Fig. 2F). Furthermore, PK5-RL-Gal-3C treatment exhibited stronger anti-tumor effect and longer survival time compared to sorafenib under the indicated conditions (Fig. 2D-F). In addition, our IHC results implied that Ki-67, which is widely used as a cell proliferation marker [21], was markedly down-regulated by treatment of PK5-RL-Gal-3C compared to negative control (Fig. 2G and H), which also confirmed the inhibitory activity of our novel fusion protein on tumor growth in vivo.

Furthermore, to further investigate the safety of our treatment, body weight of tumor-bearing mice was measured every three days, which showed that all treatment did not affect body weight and health conditions of the mice (Fig. S2). Moreover, HE staining results of internal organs including heart, liver, spleen, lung and kidney from tumor-bearing mice implied that all treatment did not cause obvious internal organ damages (Fig. S3).

### PK5-RL-Gal-3C inhibits angiogenesis in vitro and in vivo

**Fig. 3:**
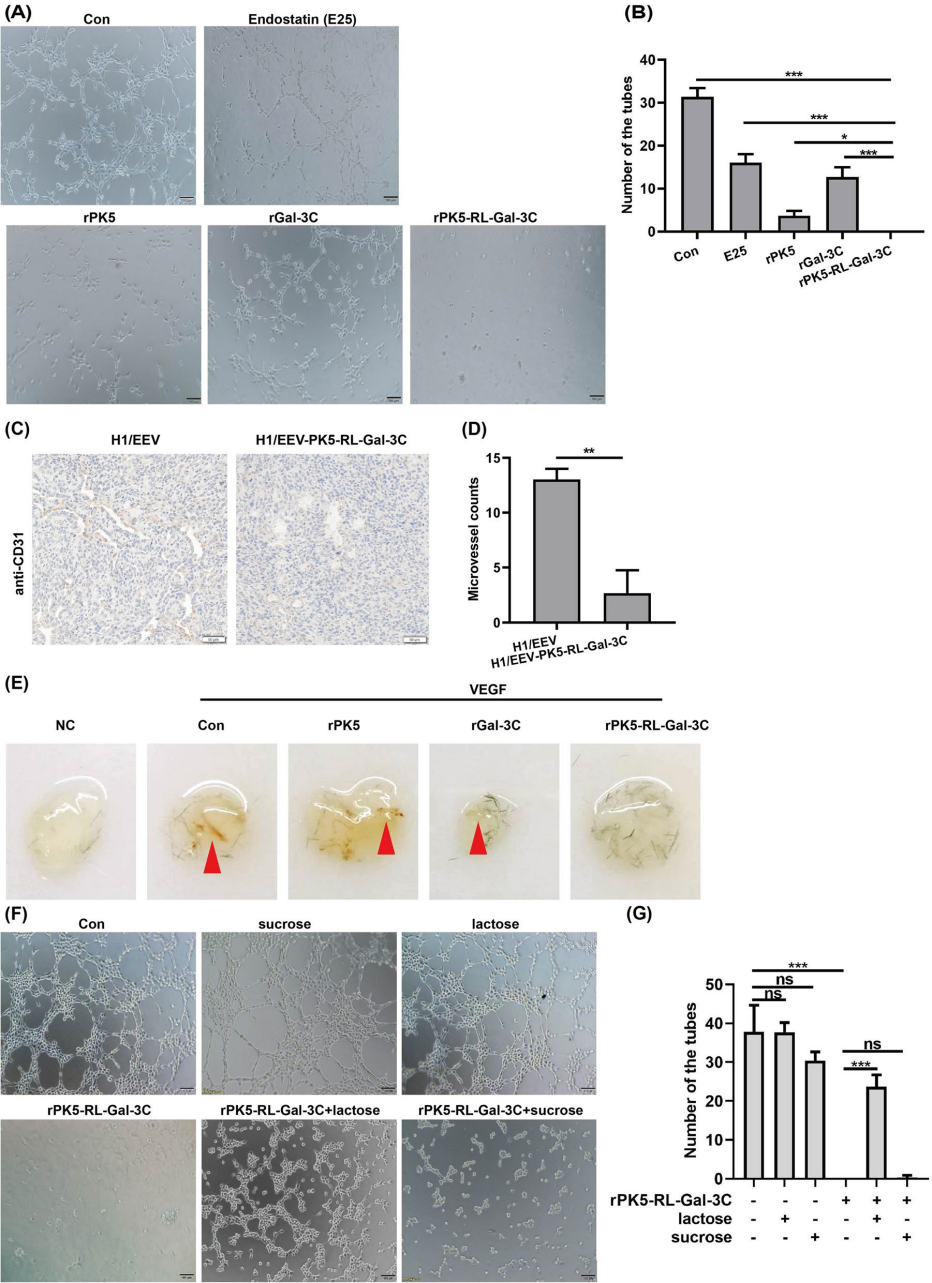
PK5-RL-Gal-3C inhibits angiogenesis in vivo and in vitro. Tube formation assay in vitro was performed to determine the inhibitory effect of rPK5-RL-Gal-3C on angiogenesis according to the materials and methods. **A** and **B** rPK5-RL-Gal-3C exhibited stronger inhibitory action than E25, rPK5 and rGal-3C on tube formation in vitro. After treated with H1/EEV and H1/EEV-PK5-RL-Gal-3C nanoparticles, the tumors were removed and stained by CD31 antibody using IHC. **C** and **D** H1/EEV-PK5-RL-Gal-3C treatment down-regulated the expression of CD31 in tumor tissues. VEGF-induced matrigel plug assay in vivo were performed to determine the inhibitory effect of rPK5-RL-Gal-3C on angiogenesis according to the materials and methods. **E** rPK5-RL-Gal-3C exhibited stronger inhibitory action than rPK5 and rGal-3C in VEGF-induced matrigel plug assay model in vivo. **F** and **G** lactose partially blockaded the inhibitory action of rPK5-RL-Gal-3C but not sucrose. Significant differences are denoted by * for* p* < 0.05, ** for *p* < 0.01, *** for *p* < 0.001 and ns, no significance

Previous investigations have reported that both PK5 and Gal-3C could inhibit angiogenesis [11, 22]. Accordingly, we hypothesized that our fusion proteins rPK5-RL-Gal-3C probably exhibited better anti-angiogenesis effect to inhibit tumor growth. In order to confirm this hypothesis, tube formation analysis in vitro was performed. E25, N-terminal 25 amino acid residues of human endostatin, was used as positive control in the present study. As shown in Fig. 3A and B, E25 could partially inhibit tube formation compared to the negative control. Surprisingly, rPK5-RL-Gal-3C completely abolished tube formation compared to rPK5, rGal-3C, and even positive control—E25 at the equimolar concentration (2 μmol/l), which suggested that rPK5-RL-Gal-3C possessed stronger anti-angiogenesis effect in vitro.

In order to further determine the inhibitory effect of PK5-RL-Gal-3C on angiogenesis in vivo, IHC was performed on tumor tissues derived from tumor-bearing mice. As shown in Fig. 3C and D, PK5-RL-Gal3C treatment significantly decreased the expression of CD31 (the marker of new blood vessels) compared to the control, which demonstrated that PK5-RL-Gal3C could inhibit angiogenesis in vivo. VEGF is a key driver of angiogenesis and highly expressed in most solid tumors [23]. Therefore, VEGF-induced matrigel plug model was also chosen to investigate the anti-angiogenic activity of rPK5-RL-Gal-3C in vivo. Our results showed that VEGF could successfully induce new blood vessel formation in matrigel plug compared to the negative control. Interestingly, rPK5 and rGal-3C could partially inhibit new blood vessel formation, but rPK5-RL-Gal-3C could obviously inhibit new blood vessel formation induced by VEGF (Fig. 3E). These results revealed that rPK5-RL-Gal-3C presented better inhibitory activity on angiogenesis in vivo, which might play important roles on their anti-tumor activity. In summary, our results demonstrated that PK5-RL-Gal-3C could significantly inhibit angiogenesis both in vitro and in vivo.

### PK5-RL-Gal-3C exhibits anti-angiogenesis activity depending on PK5 and Gal-3C domain

In order to further identify the anti-angiogenic property of novel fusion protein being mediated by PK5 or Gal-3C domain, the effect of competitive sugars on tube formation was evaluated. As shown in Fig. 3F and G, lactose (specific disaccharide inhibitor of Gal-3C) could restore 62.1% inhibition of rPK5-RL-Gal-3C on tube formation compared to lactose treatment but not sucrose. These results implied that the inhibitory effect of novel fusion protein is dependent on both Gal-3C and PK5 domains.

### PK5-RL-Gal-3C down-regulates VEGF, Ang-2, and HIF-1α in vitro and in vivo

**Fig. 4:**
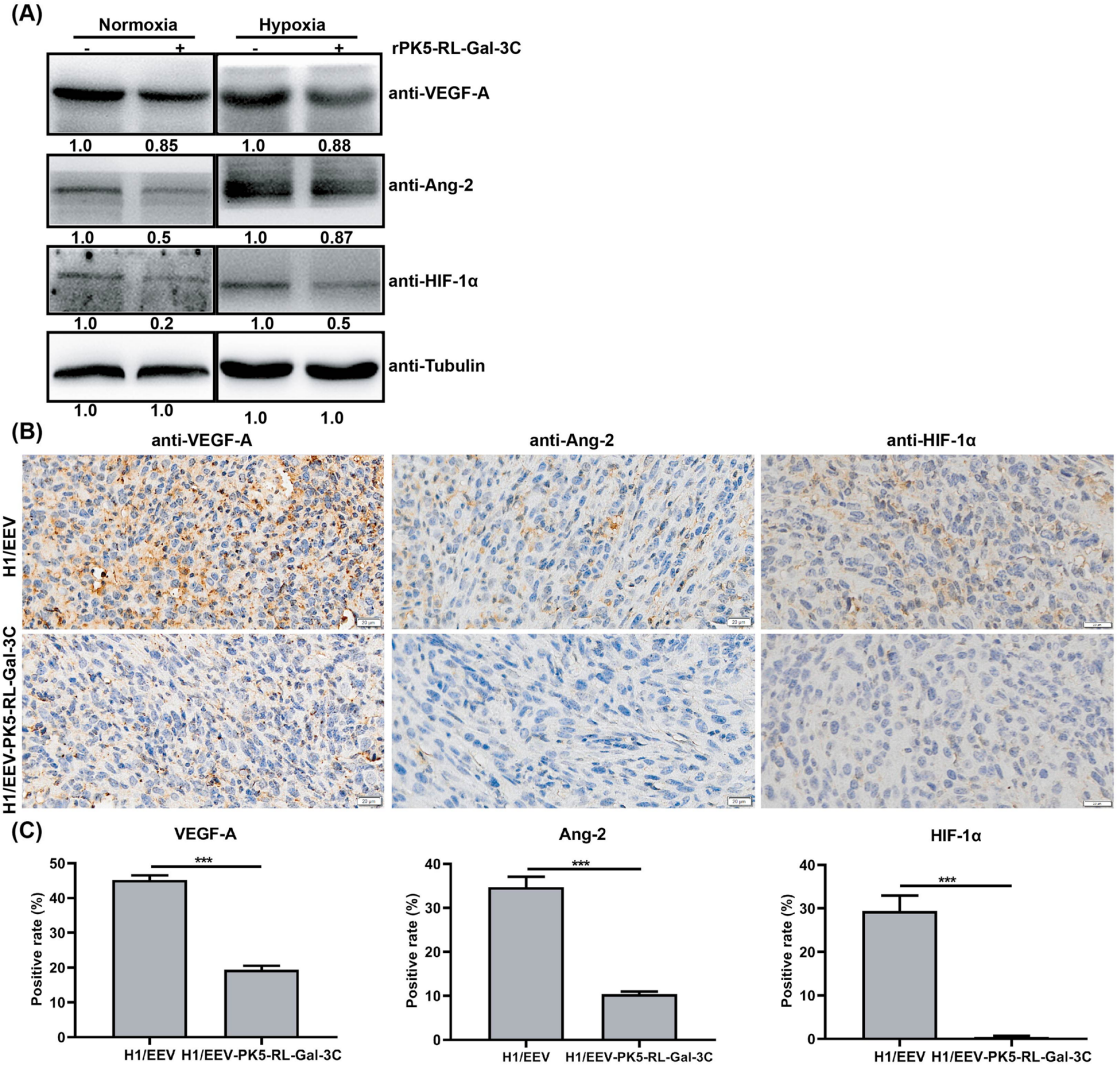
PK5-RL-Gal-3C down-regulates VEGF, Ang-2, and HIF-1α in vitro and in vivo. Huh7 cells were treated with 4 μmol/l rPK5-RL-Gal-3C for 48 h and then analyzed for differential protein expression by using western-blotting. **A** rPK5-RL-Gal-3C treatment down-regulated the expression of VEGF-A, Ang-2 and HIF-1α in Huh7 cells. Tumor tissues treated with H1/EEV-PK5-RL-Gal-3C were analyzed with IHC staining. **B** and **C** H1/EEV-PK5-RL-Gal-3C treatment down-regulated the expression of VEGF-A, Ang-2, and HIF-1α in tumor tissues. Scale bar = 20 μm. Significant differences are denoted by *** for *p* < 0.001

As known, tumor angiogenesis relies on the up-regulation of pro-angiogenic factors [2]. Our western-blotting results presented that rPK5-RL-Gal-3C treatment could decrease the expression of VEGF-A and Ang-2 in Huh7 cells under normoxia and hypoxia (DFO induced) conditions (Fig. 4A). Additionally, our IHC results also showed that VEGF-A and Ang-2 were significantly down-regulated in xenograft HCC tumor tissues after treated with PK5-RL-Gal-3C in vivo (Fig. 4B and C). All these results suggested that PK5-RL-Gal-3C might blockade VEGF-A and Ang-2 expression in HCC to inhibit angiogenesis in a paracrine manner.

HIF-1α, as the most important hypoxic response factor, could regulate cancer angiogenesis as transcriptional factor via promoting its major target gene VEGF-A. It has been demonstrated that PK5 could promote SUMO/ubiquitin-mediated proteasomal degradation of HIF-1α to inhibit VEGF expression in gastric cancer cells under hypoxia [15]. Our current studies also showed that PK5-RL-Gal-3C could down-regulate the expression of HIF-1α in Huh7 cells under normoxia and even hypoxia conditions (Fig. 4A). Furthermore, our IHC staining results confirmed that PK5-RL-Gal-3C treatment could decrease the expression of HIF-1α in xenograft HCC tumor tissues in vivo (Fig. 4B and C). All these results implied that PK5-RL-Gal-3C could inhibit hypoxia-induced angiogenesis under tumor microenvironments.

### PK5-RL-Gal-3C inhibits HCC cell proliferation depending on Gal-3C domain in vitro

**Fig. 5:**
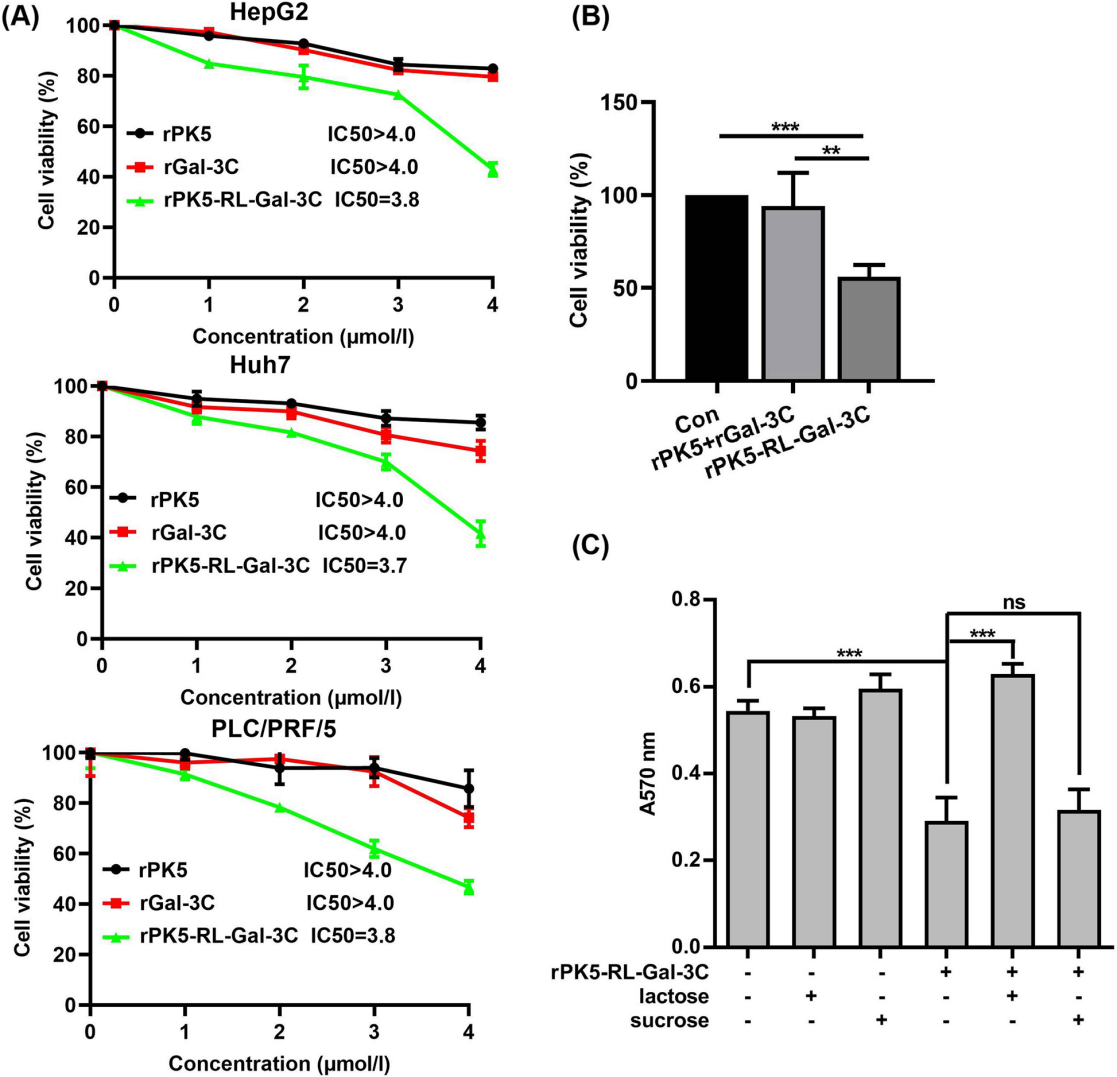
PK5-RL-Gal-3C inhibits HCC cell proliferation in vitro. HCC cells were respectively treated with rPK5, rGal-3C, and rPK5-RL-Gal-3C under the indicated concentrations (0, 1, 2, 3 and 4 μmol/l) for 48 h, and then analyzed with a MTT assay kit. **A** rPK5-RL-Gal-3C treatment inhibited cell proliferation of HepG2, Huh7, and PLC/PRF/5. **B** rPK5-RL-Gal-3C exhibited stronger inhibitory action than combination of rPK5 and rGal-3C to PLC/PRF/5 cells. **C** Lactose not sucrose could suppress the inhibitory effect of rPK5-RL-Gal-3C to PLC/PRF/5 cells. Significant differences are denoted by ** for *p* < 0.01, *** for *p* < 0.001, and ns, no significance

In order to further investigate the anti-tumor effects of rPK5-RL-Gal-3C in vitro, HCC cell proliferation analysis was analyzed. After treated with rPK5, rGal-3C, and rPK5-RL-Gal-3C under the increasing concentrations (0, 1, 2, 3, 4 μmol/l) for 48 h, cell proliferation was detected with MTT assay kit. As shown in Fig. 5A, rPK5 and rGal-3C exhibited weak inhibition on HepG2, Huh7, and PLC/PRF/5 cells with IC_50_ value of more than 4 μmol/l, which is consistent with the results reported previously [10, 24]. Surprisingly, rPK5-RL-Gal3C could inhibit HepG2, Huh7, and PLC/PRF/5 cells proliferation with similar IC_50_ value of 3.8, 3.7, and 3.8 μmol/l, respectively.

In order to investigate the inhibitory effect of rPK5-RL-Gal-3C on tumor cell proliferation derived from synergy or combination of PK5 and Gal-3C domains, we further compared the inhibitory action between rPK5-RL-Gal-3C and combined rPK5 with rGal-3C (rPK5 + rGal-3C) at the same molar concentration (4 μmol/l). As shown in Fig. 5B, combined rPK5 with rGal-3C exhibited weak inhibition on PLC/PRF/5 cell proliferation, but rPK5-RL-Gal-3C exhibited stronger inhibition, which demonstrated that the inhibitory action of rPK5-RL-Gal-3C on HCC cells is complicated synergy but not simple combination of PK5 and Gal-3C domains.

Previous studies have revealed that PK5 could inhibit tumor cell proliferation in hypoxia but not in normoxia [15], while Gal-3C could inhibit HCC cell proliferation in normoxia [10]. In our present study, in order to characterize the cell proliferation inhibitory action of rPK5-RL-Gal-3C mediated by PK5 and/or Gal-3C domain, lactose was introduced to blockade the activity of Gal-3C domain. Our results showed that presence of lactose could almost completely abolish the inhibition of PK5-RL-Gal-3C on HCC cell proliferation but not sucrose (Fig. 5C), which suggested that rPK5-RL-Gal-3C could target HCC cell proliferation in a Gal-3C-dependent manner, and PK5 enhanced its inhibitory function.

### PK5-RL-Gal-3C induces G1 cell cycle arrest of HCC in vitro

**Fig. 6:**
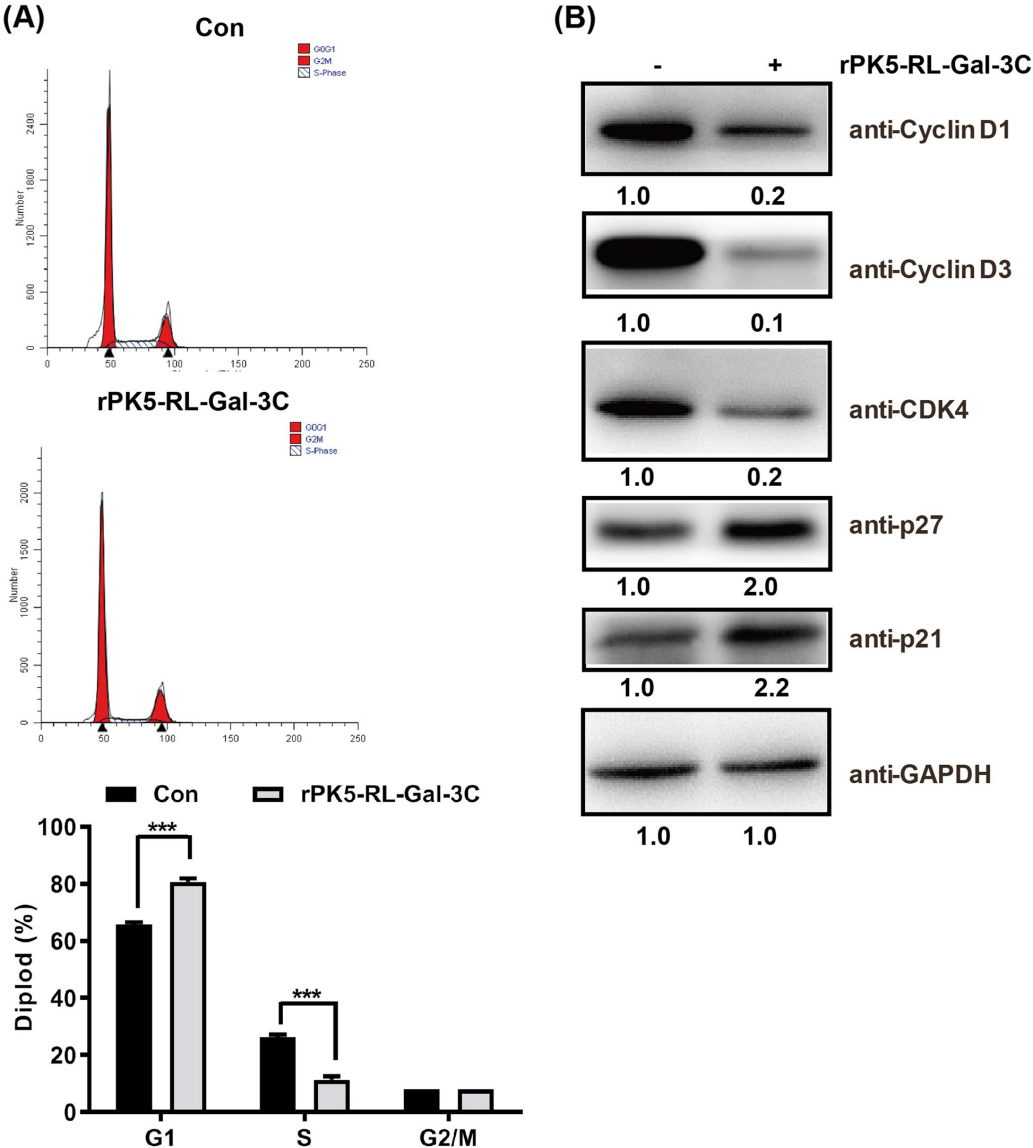
PK5-RLGal-3C induces G1 cell cycle arrest. After treated with 4 μmol/l rPK5-RL-Gal-3C for 48 h, HepG2 cells were stained with PI and then analyzed with flow cytometer. **A** rPK5-RL-Gal-3C treatment induced G1 cell cycle arrest on HepG2 cells. Western-blotting was performed to analyze the expression of proteins associated with cell cycle arrest. **B** rPK5-RL -Gal-3C treatment down-regulated the protein expression of Cyclin D1, Cyclin D3, and CDK4, but up-regulated the protein expression of p21 and p27. Significant differences are denoted by *** for *p* < 0.001

In order to further explore the inhibition of rPK5-RL-Gal-3C on cell proliferation, cell cycle was determined. After treated with rPK5-RL-Gal-3C for 48 h, HepG2 cells were stained with PI and analyzed by flow cytometer. Our data presented that rPK5-RL-Gal-3C treatment could significantly induce G1 cell cycle arrest on HepG2 cells (Fig. 6A). Furthermore, western-blotting assay was carried out to determine the protein expression associated with cell cycle. Our results showed that the expression of Cyclin D1, Cyclin D3, and CDK4, which are important regulatory factors promoting cell cycle transition from G1 to S phase, was markedly down-regulated in HepG2 cells following treated by rPK5-RL-Gal-3C for 48 h (Fig. 6B). Moreover, the expression of p27 and p21 was up-regulated, both of which are well-known inhibitors of Cyclin D1-CDK4 complex. These results suggested that cell cycle arrest was involved in the inhibition of HCC cell proliferation induced by rPK5-RLGal-3C.

### PK5-RL-Gal-3C induces cell apoptosis of HCC in vitro

**Fig. 7:**
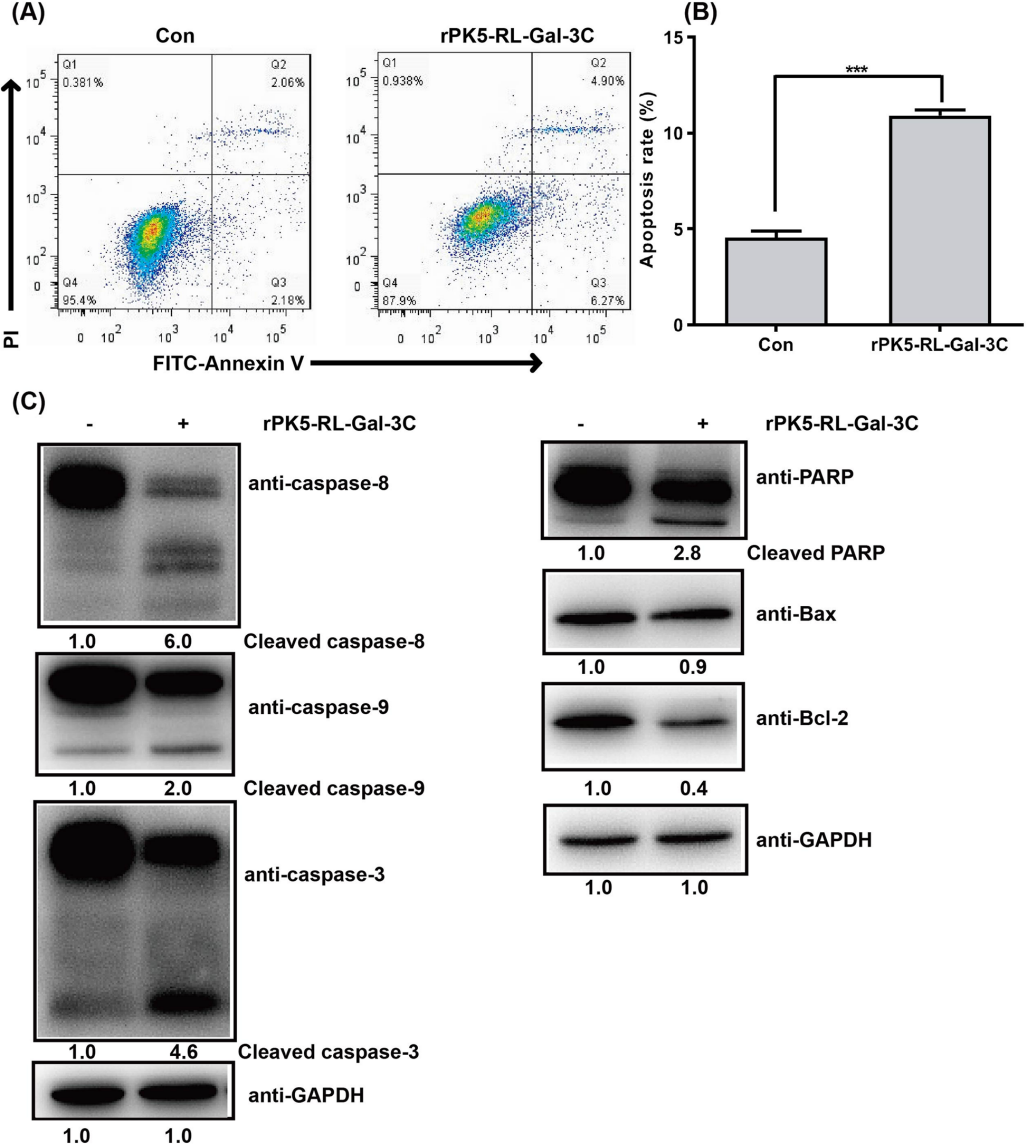
PK5-RLGal-3C induces cell apoptosis. After treated with 4 μmol/l rPK5-RL-Gal-3C for 48 h, HepG2 cells were stained with FITC-Annexin V/PI and then analyzed with flow cytometer. **A** and **B** rPK5-RL-Gal-3C treatment induced cell apoptosis on HepG2 cells. Western-blotting was performed to analyze the expression of proteins associated with cell apoptosis. **C** rPK5-RL-Gal-3C treatment induced protein expression of cleaved caspase-8, caspase-9, caspase-3, PARP, but down-regulated anti-apoptotic protein expression of Bcl-2. Significant differences are denoted by *** for *p* < 0.001

Cell cycle arrest and apoptosis are the most common reasons for the inhibition of cell growth. After treated by rPK5-RL-Gal-3C for 48 h, HepG2 cells were stained with FITC-Annexin V/PI cell apoptosis kit and then analyzed by flow cytometer. Our results showed that rPK5-RL-Gal-3C treatment could also induce apoptosis of HepG2 cells (10.9%) (Fig. 7A and B). To obtain further insight into mechanisms of rPK5-RL-Gal-3C induced cell apoptosis, western-blotting assay was carried out to determine the protein expression associated with cell apoptosis. rPK5-RL-Gal-3C treatment also significantly induced cleavage of caspase-3 and PARP, which are important marker of apoptosis and able to inhibit DNA damage repair (Fig. 7C). Interestingly, the treatment of rPK5-RL-Gal-3C could also obviously down-regulate the expression of Bcl-2, a well-known anti-apoptotic protein. The expression of pro-apoptotic protein Bax did not change. Whereas, the expression of cleaved caspase-8 and caspase-9 was up-regulated, which implied that rPK5-RL-Gal-3C treatment might activate both extrinsic and intrinsic apoptosis pathways (Fig. 7C).

## Discussion

In recent years, published reports have proved that both Gal-3N-mediated oligomerization and Gal-3C-mediated carbohydrate binding activity are required for endogenous Gal-3 mediated angiogenesis [11]. Although Gal-3C contains contact carbohydrate recognition domain and retains its ability to bind to β-galactoside-containing glycans, it is unable to form dimer or oligomer because of lacking Gal-3N [25]. Moreover, Gal-3C, as dominant-negative inhibitor of full-length Gal-3, presented potential anti-tumor activity via inhibiting angiogenesis, reducing tumor growth and metastasis [10, 12, 26, 27]. Thus, the specific target and inhibition of Gal-3C impulse us to hypothesize for the first time that introduction of functional domain to the N-terminus of Gal-3C would enhance its anti-tumor activity.

Kringle, composed of appropriately 80 amino acids, is a triple loop structure and linked by three pairs of disulfide bonds formed by six conserved cysteine residues [28]. Several studies have indicated that the kringle domain has potent anti-angiogenic activity although derived from different proteins, such as plasminogen [13], hepatocyte growth factor [20], and apolipoprotein [29]. In the previous studies, we have demonstrated anti-tumor activity of the first kringle domain from hepatocyte growth factor (HGFK1) in glioblastoma [30] and renal cell carcinoma [20]. It is well known that PK5 is the most active anti-angiogenic factor of all plasminogen proteolytic fragments by specifically inhibiting cell growth, migration, and following angiogenesis in proliferating endothelial cells [13, 31].

In the present study, PK5 was introduced to the N-terminus of Gal-3C via RL to generate novel fusion protein PK5-RL-Gal-3C, which has similar chimeric structure and carbohydrate binding activity with wild type Gal-3. Interestingly, the fusion protein PK5-RL-Gal-3C exhibits more potent anti-tumor activity than PK5 and Gal-3C via inhibiting angiogenesis and tumor cell proliferation both in vivo and in vitro. Importantly, both PK5 and Gal-3C domains are derived from endogenous proteolytic fragment with less immunogenicity and toxicity to the host. Concomitantly, PK5-RL-Gal-3C treatment did not cause obvious cytotoxic side effect in vivo.

Previous studies have reported that PK5 prefers to suppress tumor cells under hypoxic but not normoxic conditions because of higher expression of its ligands GRP78 and VDAC1 on tumor cells under hypoxic conditions [15]. Our results also indicated that PK5 did not exhibit obvious inhibitory effect on HCC cell proliferation in vitro, but it was involved in anti-angiogenic activity of the fusion protein. In addition, it has been demonstrated that Gal-3C could inhibit endogenous Gal-3 induced angiogenesis and blockade tumor cell proliferation, migration, and invasion [10–12, 26, 27]. In the present study, our data showed that Gal-3C participated both anti-angiogenesis and cytotoxicity of the novel fusion protein to HCC. Therefore, the anti-tumor activity of PK5-RL-Gal-3C is synergistic function derived from PK5 and Gal-3C.

Angiogenesis is a complicated progress with a series of steps, such as activation of endothelial cells, degradation of basement membranes, endothelial cell proliferation, migration, and new blood tubule formation [2]. It has been reported that many angiogenic pathways are involved in the progression of HCC [32]. Therefore, partial cleavage of Gal-3N could enhance the interaction of truncated Gal-3 with endothelial cells and improve pro-angiogenic activity of Gal-3, while complete cleavage of Gal-3N and only remaining Gal-3C exhibits anti-angiogenic activity; which indicated that oligomerization is involved in full-length Gal-3 induced angiogenesis [11, 33]. In this study, the novel fusion protein only retains Gal-3C domain to inhibit angiogenesis, which could be blockaded by competitive disaccharide—lactose but not sucrose. And the addition of PK5 domain further enhanced the anti-angiogenic activity of the fusion protein PK5-RL-Gal-3C.

Until now, several studies have reported that single anti-VEGF therapy often develop rapid drug resistance, owing to complementary activation of other pro-angiogenic signaling pathways, *e. g.*, Ang-2 that is a potential driver of resistance to VEGF inhibition [34, 35]. Moreover, it has also been demonstrated that dual inhibition of VEGF and Ang-2 could delay tumor growth and prolong survival time in glioblastoma [35, 36]. VDAC1 and GRP78 have been identified as receptors for PK5 to mediate anti-angiogenesis by down-regulating HIF-1α/VEGF, and induce tumor cell apoptosis in hypoxic condition [15, 37, 38]. Our further mechanism studies implied that PK5-RL-Gal-3C treatment could down-regulate HIF-1α and VEGF in HCC cells. Besides, PK5-RL-Gal-3C treatment could also decrease the expression of Ang-2. All of these results revealed that our fusion proteins could inhibit both VEGF and Ang-2 pathways to inhibit angiogenesis.

New tumor blood vessels, formed under the imbalance between pro-angiogenic and anti-angiogenic factors, are structural and functional abnormal with excessive permeability, poor perfusion and increased hypoxia that promote tumor growth and metastasis, as well as affect immune cells activation and infiltration [39, 40]. Increasing evidences have demonstrated that anti-angiogenesis therapy could improve anti-tumor immunity [39, 41]. Therefore, PK5-RL-Gal-3C treatment might enhance the immune cell anti-tumor activity by inhibiting angiogenesis that should be evaluated by further investigations in the following studies. Besides in tumor, PK5-RL-Gal-3C would play potential roles via inhibiting angiogenesis in the treatment of angiogenesis associated diseases such as diabetic retinopathy, rheumatoid arthritis, and would healing.

In our present study, rPK5-RL-Gal-3C was purified via α-lactose-agarose that implied that rPK5-RL-Gal-3C is the potential competitively antagonist of endogenous full-length Gal-3. In addition, the results showed that PK5-RL-Gal-3C could inhibit HCC cell proliferation in vitro by significantly inducing cell cycle arrest and minor apoptosis (< 10% apoptosis rate), which are the most common pathways associated with cell proliferation. p21 and p27 are negative regulators of cell cycle progression, and also involved in apoptosis, transcription, as well as DNA damage [42]. Previous studies showed that Gal-3 could promote cell growth and exhibit anti-apoptotic activity by stabilizing the expression of p21 via its Gal-3C domain in prostate cancer [42]. Moreover, specific inhibition of Gal-3 with antagonist GCS-100/modified citrus pectin could up-regulate p21 and induce G1 cell cycle arrest and apoptosis in myeloma [43]. In addition, ablation of Gal-3 could induce G1 cell cycle arrest in p27-dependent manner to inhibit cell proliferation by inducing premature senescence in gastric cancer [44]. In the present study, we demonstrated that PK5-RL-Gal-3C could increase the expression of p21 and p27 to inhibit cell proliferation by inducing G1 cell cycle arrest and apoptosis accompanying with decreased the protein expression of cyclin D and CDK4 that might be associate with the inhibition of endogenous Gal-3.

It is well established that anti- and pro-apoptotic activities of Gal-3 is tightly associated with its localization [7]. Generally, intracellular Gal-3 could protect tumor cells against cell apoptosis induced by various stimuli. And several signaling pathways are involved in Gal-3 anti-apoptotic activity, such as preventing mitochondrial damage, inhibiting cytochrome c release, binding with anti-apoptotic protein Bcl-2 via Gal-3C domain [7]. In the present study, PK5-RL-Gal-3C treatment could induce mitochondria-dependent intrinsic apoptosis pathway and down-regulate the expression of Bcl-2, which suggested that the fusion protein might be internalized into cell cytoplasm to inhibit intracellular full-length Gal-3. Additionally, caspased-8 cleavage indicated that PK5-RL-Gal-3C treatment activated extrinsic apoptosis pathway, but the receptor remains unknown, which should be further investigated in the future.

In summary, our results reveal the potent anti-tumor activity and mechanisms of fusion protein PK5-RL-Gal-3C in HCC. And, endogenous full-length Gal-3 is the potential target of PK5-RL-Gal-3C. All the data provide new strategy for exploring novel antagonist of Gal-3 and promotes their application in clinical treatment.

## Supplementary Information

**Additional file 1**.

## Data Availability

The datasets analyzed in the current study are available on request to the corresponding author.

## Abbreviations

Ang-2: Angiopoietin-2
DFO: Deferoxamine
Gal-3: Galectin-3
Gal-3C: Gal-3 C-terminal carbohydrate-recognition domain
GRP78: Glucose-regulated protein 78
HCC: Hepatocellular carcinoma
HIF-1α: Hypoxia-inducible factor 1α
PK5: The fifth kringle domain of plasminogen
PDGFR: Platelet-derived growth factor receptor
RL: Rigid linker
VDAC1: Voltage-dependent anion channel 1
VEGF: Vascular endothelial growth factor

## References

[1] Ramjiawan RR, Griffioen AW, Duda DG (2017). Anti-angiogenesis for cancer revisited: is there a role for combinations with immunotherapy?. Angiogenesis.

[2] Viallard C, Larrivee B (2017). Tumor angiogenesis and vascular normalization: alternative therapeutic targets. Angiogenesis.

[3] Jain RK (2014). Antiangiogenesis strategies revisited: from starving tumors to alleviating hypoxia. Cancer Cell.

[4] Villanueva A (2019). Hepatocellular Carcinoma. N Engl J Med.

[5] Thomas H (2018). Liver cancer: Lenvatinib non-inferior to sorafenib for hepatocellular carcinoma. Nat Rev Gastroenterol Hepatol.

[6] Simon T, Gagliano T, Giamas G (2017). Direct effects of anti-angiogenic therapies on tumor cells: VEGF signaling. Trends Mol Med.

[7] Dumic J, Dabelic S, Flogel M (2006). Galectin-3: an open-ended story. Biochim Biophys Acta.

[8] Fortuna-Costa A, Gomes AM, Kozlowski EO, Stelling MP, Pavao MS (2014). Extracellular galectin-3 in tumor progression and metastasis. Front Oncol.

[9] Funasaka T, Raz A, Nangia-Makker P (2014). Galectin-3 in angiogenesis and metastasis. Glycobiology.

[10] Wang M, Tian F, Ying W, Qian X (2017). Quantitative proteomics reveal the anti-tumour mechanism of the carbohydrate recognition domain of Galectin-3 in hepatocellular carcinoma. Sci Rep.

[11] Markowska AI, Liu FT, Panjwani N (2010). Galectin-3 is an important mediator of VEGF- and bFGF-mediated angiogenic response. J Exp Med.

[12] John CM, Leffler H, Kahl-Knutsson B, Svensson I, Jarvis GA (2003). Truncated galectin-3 inhibits tumor growth and metastasis in orthotopic nude mouse model of human breast cancer. Clin Cancer Res.

[13] Cao Y, Chen A, An SS, Ji RW, Davidson D, Llinas M (1997). Kringle 5 of plasminogen is a novel inhibitor of endothelial cell growth. J Biol Chem.

[14] Yang X, Cheng R, Li C, Cai W, Ma JX, Liu Q, Yang Z, Song Z, Liu Z, Gao G (2006). Kringle 5 of human plasminogen suppresses hepatocellular carcinoma growth both in grafted and xenografted mice by anti-angiogenic activity. Cancer Biol Ther.

[15] Fang S, Hong H, Li L, He D, Xu Z, Zuo S, Han J, Wu Q, Dai Z, Cai W, Ma J, Shao C, Gao G, Yang X (2017). Plasminogen kringle 5 suppresses gastric cancer via regulating HIF-1alpha and GRP78. Cell Death Dis.

[16] Jin GH, Ma DY, Wu N, Marikar FM, Jin SZ, Jiang WW, Liu Y, Hua ZC (2007). Combination of human plasminogen kringle 5 with ionizing radiation significantly enhances the efficacy of antitumor effect. Int J Cancer.

[17] Hsieh JL, Wu CL, Lee CH, Shiau AL (2003). Hepatitis B virus X protein sensitizes hepatocellular carcinoma cells to cytolysis induced by E1B-deleted adenovirus through the disruption of p53 function. Clin Cancer Res.

[18] Yao H, Ng SS, Tucker WO, Tsang YK, Man K, Wang XM, Chow BK, Kung HF, Tang GP, Lin MC (2009). The gene transfection efficiency of a folate-PEI600-cyclodextrin nanopolymer. Biomaterials.

[19] Gao X, Wei M, Shan W, Liu Q, Gao J, Liu Y, Zhu S, Yao H (2019). An oral 2-hydroxypropyl-beta-cyclodextrin-loaded spirooxindole-pyrrolizidine derivative restores p53 activity via targeting MDM2 and JNK1/2 in hepatocellular carcinoma. Pharmacol Res.

[20] Gao X, Jiang P, Zhang Q, Liu Q, Jiang S, Liu L, Guo M, Cheng Q, Zheng J, Yao H (2019). Peglated-H1/pHGFK1 nanoparticles enhance anti-tumor effects of sorafenib by inhibition of drug-induced autophagy and stemness in renal cell carcinoma. J Exp Clin Cancer Res.

[21] Sun X, Kaufman PD (2018). Ki-67: more than a proliferation marker. Chromosoma.

[22] Gao G, Li Y, Gee S, Dudley A, Fant J, Crosson C, Ma JX (2002). Down-regulation of vascular endothelial growth factor and up-regulation of pigment epithelium-derived factor: a possible mechanism for the anti-angiogenic activity of plasminogen kringle 5. J Biol Chem.

[23] Goel HL, Mercurio AM (2013). VEGF targets the tumour cell. Nat Rev Cancer.

[24] Davidson DJ, Haskell C, Majest S, Kherzai A, Egan DA, Walter KA, Schneider A, Gubbins EF, Solomon L, Chen Z, Lesniewski R, Henkin J (2005). Kringle 5 of human plasminogen induces apoptosis of endothelial and tumor cells through surface-expressed glucose-regulated protein 78. Cancer Res.

[25] Ahmad N, Gabius HJ, Andre S, Kaltner H, Sabesan S, Roy R, Liu B, Macaluso F, Brewer CF (2004). Galectin-3 precipitates as a pentamer with synthetic multivalent carbohydrates and forms heterogeneous cross-linked complexes. J Biol Chem.

[26] Mirandola L, Yu Y, Cannon MJ, Jenkins MR, Rahman RL, Nguyen DD, Grizzi F, Cobos E, Figueroa JA, Chiriva-Internati M (2014). Galectin-3 inhibition suppresses drug resistance, motility, invasion and angiogenic potential in ovarian cancer. Gynecol Oncol.

[27] Mirandola L, Yu Y, Chui K, Jenkins MR, Cobos E, John CM, Chiriva-Internati M (2011). Galectin-3C inhibits tumor growth and increases the anticancer activity of bortezomib in a murine model of human multiple myeloma. PLoS One.

[28] Cao Y, Cao R, Veitonmaki N (2002). Kringle structures and antiangiogenesis. Curr Med Chem Anticancer Agents.

[29] Kim JS, Chang JH, Yu HK, Ahn JH, Yum JS, Lee SK, Jung KH, Park DH, Yoon Y, Byun SM, Chung SI (2003). Inhibition of angiogenesis and angiogenesis-dependent tumor growth by the cryptic kringle fragments of human apolipoprotein(a). J Biol Chem.

[30] Zhang W, Duan R, Zhang J, Cheung WKC, Gao X, Zhang R, Zhang Q, Wei M, Wang G, Zhang Q, Mei PJ, Chen HL, Kung H, Lin MC, Shen Z, Zheng J, Zhang L, Yao H (2018). H1/pHGFK1 nanoparticles exert anti-tumoural and radiosensitising effects by inhibition of MET in glioblastoma. Br J Cancer.

[31] Nguyen TM, Subramanian IV, Kelekar A, Ramakrishnan S (2007). Kringle 5 of human plasminogen, an angiogenesis inhibitor, induces both autophagy and apoptotic death in endothelial cells. Blood.

[32] Morse MA, Sun W, Kim R, He AR, Abada PB, Mynderse M (2019). The role of angiogenesis in hepatocellular carcinoma. Clin Cancer Res.

[33] Nangia-Makker P, Wang Y, Raz T, Tait L, Balan V, Hogan V, Raz A (2010). Cleavage of galectin-3 by matrix metalloproteases induces angiogenesis in breast cancer. Int J Cancer.

[34] Chae SS, Kamoun WS, Farrar CT, Kirkpatrick ND, Niemeyer E, de Graaf AM, Sorensen AG, Munn LL, Jain RK, Fukumura D (2010). Angiopoietin-2 interferes with anti-VEGFR2-induced vessel normalization and survival benefit in mice bearing gliomas. Clin Cancer Res.

[35] Peterson TE, Kirkpatrick ND, Huang Y, Farrar CT, Marijt KA, Kloepper J, Datta M, Amoozgar Z, Seano G, Jung K, Kamoun WS, Vardam T, Snuderl M, Goveia J, Chatterjee S, Batista A, Muzikansky A, Leow CC, Xu L, Batchelor TT, Duda DG, Fukumura D, Jain RK (2016). Dual inhibition of Ang-2 and VEGF receptors normalizes tumor vasculature and prolongs survival in glioblastoma by altering macrophages. Proc Natl Acad Sci USA.

[36] Kloepper J, Riedemann L, Amoozgar Z, Seano G, Susek K, Yu V, Dalvie N, Amelung RL, Datta M, Song JW, Askoxylakis V, Taylor JW, Lu-Emerson C, Batista A, Kirkpatrick ND, Jung K, Snuderl M, Muzikansky A, Stubenrauch KG, Krieter O, Wakimoto H, Xu L, Munn LL, Duda DG, Fukumura D, Batchelor TT, Jain RK (2016). Ang-2/VEGF bispecific antibody reprograms macrophages and resident microglia to anti-tumor phenotype and prolongs glioblastoma survival. Proc Natl Acad Sci USA.

[37] Dong D, Ko B, Baumeister P, Swenson S, Costa F, Markland F, Stiles C, Patterson JB, Bates SE, Lee AS (2005). Vascular targeting and antiangiogenesis agents induce drug resistance effector GRP78 within the tumor microenvironment. Cancer Res.

[38] Gonzalez-Gronow M, Kalfa T, Johnson CE, Gawdi G, Pizzo SV (2003). The voltage-dependent anion channel is a receptor for plasminogen kringle 5 on human endothelial cells. J Biol Chem.

[39] Fukumura D, Kloepper J, Amoozgar Z, Duda DG, Jain RK (2018). Enhancing cancer immunotherapy using antiangiogenics: opportunities and challenges, Nature reviews. Clin Oncol.

[40] Hendry SA, Farnsworth RH, Solomon B, Achen MG, Stacker SA, Fox SB (2016). The role of the tumor vasculature in the host immune response: implications for therapeutic strategies targeting the tumor microenvironment. Front Immunol.

[41] Huang Y, Goel S, Duda DG, Fukumura D, Jain RK (2013). Vascular normalization as an emerging strategy to enhance cancer immunotherapy. Cancer Res.

[42] Wang Y, Balan V, Kho D, Hogan V, Nangia-Makker P, Raz A (2013). Galectin-3 regulates p21 stability in human prostate cancer cells. Oncogene.

[43] Streetly MJ, Maharaj L, Joel S, Schey SA, Gribben JG, Cotter FE (2010). GCS-100, a novel galectin-3 antagonist, modulates MCL-1, NOXA, and cell cycle to induce myeloma cell death. Blood.

[44] Kim SJ, Lee HW, Gu Kang H, La SH, Choi IJ, Ro JY, Bresalier RS, Song J, Chun KH (2014). Ablation of galectin-3 induces p27(KIP1)-dependent premature senescence without oncogenic stress. Cell Death Differ.

