# Correction: Novel fusion protein PK5-RL-Gal-3 C inhibits hepatocellular carcinoma via anti-angiogenesis and cytotoxicity

**DOI:** 10.1186/s12885-023-10843-0

**Published:** 2023-04-19

**Authors:** Xiaoge Gao, Pin Jiang, Xiaohuan Wei, Wei Zhang, Jiwei Zheng, Shishuo Sun, Hong Yao, Xiangye Liu, Qing Zhang

**Affiliations:** 1 Cancer Institute, Xuzhou Medical University, Xuzhou, Jiangsu Province 221004 People’s Republic of China; 2 Center of Clinical Oncology, The Affiliated Hospital of Xuzhou Medical University, Xuzhou, Jiangsu Province 221004 People’s Republic of China; 3 Jiangsu Center for the Collaboration and Innovation of Cancer Biotherapy, Xuzhou Medical University, Xuzhou, Jiangsu Province 221004 People’s Republic of China; 4 Nanjing International Hospital Co., Ltd, Nanjing, Jiangsu Province 210000 People’s Republic of China; 5 Medical Oncology of Huangmei People’s Hospital, Huanggang, Hubei Province 435500 People’s Republic of China; 6 Department of Oral Medicine, School of Stomatology, Xuzhou Medical University, Xuzhou, Jiangsu 221004 People’s Republic of China; 7 Department of Cancer Biotherapy Center, Third Affiliated Hospital of Kunming Medical University, Kunming, Yunnan Province 650118 People’s Republic of China; 8 Department of Pathogenic Biology and Immunology, Jiangsu Key Laboratory of Immunity and Metabolism, Xuzhou Medical University, Xuzhou, Jiangsu Province 221004 People’s Republic of China

**Correction**: **BMC Cancer 23, 154 (2023)**

10.1186/s12885-023-10608-9

Following publication of the original article [1], the authors identified an error in Fig. 3F. The representative tube formation figure of lactose group was wrong. The correction does not have any effect on the results and conclusions of the article. The corrected Fig. 3 is given in this correction article and the original article [1] is corrected.

**Fig. 3:**
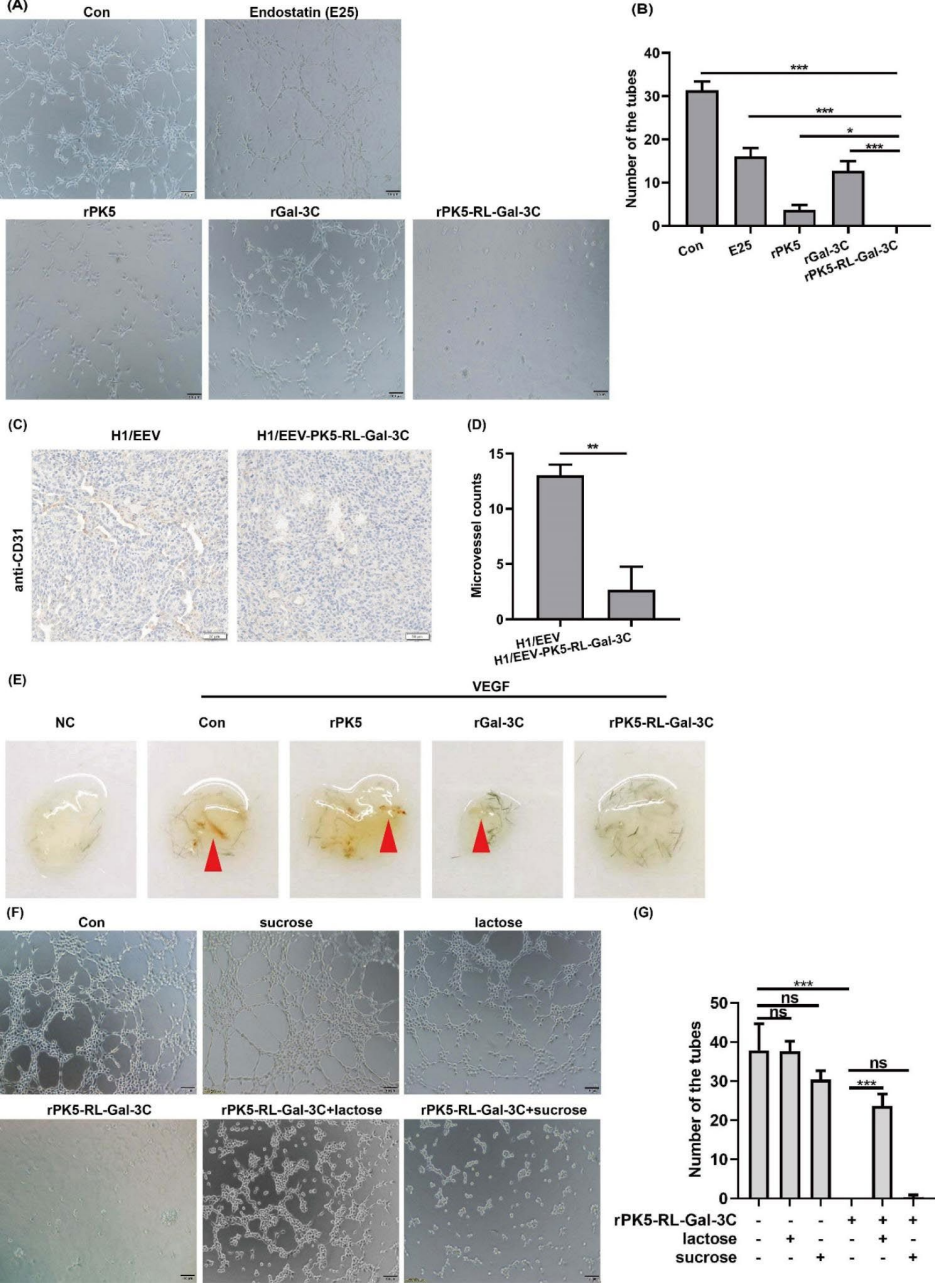
PK5-RL-Gal-3 C inhibits angiogenesis in vivo and in vitro. Tube formation assay in vitro was performed to determine the inhibitory effect of rPK5-RL-Gal-3 C on angiogenesis according to the materials and methods. **A** and **B** rPK5-RL-Gal-3 C exhibited stronger inhibitory action than E25, rPK5 and rGal-3 C on tube formation in vitro. After treated with H1/EEV and H1/EEV-PK5-RL-Gal-3 C nanoparticles, the tumors were removed and stained by CD31 antibody using IHC. **C** and **D** H1/EEV-PK5-RL-Gal-3 C treatment down-regulated the expression of CD31 in tumor tissues. VEGF-induced matrigel plug assay in vivo were performed to determine the inhibitory effect of rPK5-RL-Gal-3 C on angiogenesis according to the materials and methods. **E** rPK5-RL-Gal-3 C exhibited stronger inhibitory action than rPK5 and rGal-3 C in VEGF-induced matrigel plug assay model in vivo. **F** and **G** lactose partially blockaded the inhibitory action of rPK5-RL-Gal-3 C but not sucrose. Significant differences are denoted by * for *p* < 0.05, ** for *p* < 0.01, *** for *p* < 0.001 and ns, no significance

## References

[1] Gao X, Jiang P, Wei X (2023). Novel fusion protein PK5-RL-Gal-3 C inhibits hepatocellular carcinoma via anti-angiogenesis and cytotoxicity. BMC Cancer.

