# Supplementary material for: Novel fusion protein PK5-RL-Gal-3C inhibits hepatocellular carcinoma via anti-angiogenesis and cytotoxicity
Source: BMC Cancer. 2023 Feb 15;23:154. doi: 10.1186/s12885-023-10608-9 (PMC9930235; doi:10.1186/s12885-023-10608-9)

**Title:** Novel fusion protein PK5-RL-Gal-3C inhibits hepatocellular carcinoma via anti-angiogenesis and cytotoxicity

Xiaoge Gao^1, 2, 3, 4 *^, Pin Jiang^1, 2, 3, 5 *^, Xiaohuan Wei^1, 2, 3, 5 *^, Wei Zhang^1, 2, 3, 4^, Jiwei Zheng^6^, Shishuo Sun^1, 2, 3, 4^, Hong Yao^1, 7 #^, Xiangye Liu^8 #^, and Qing Zhang^1, 2, 3, 4 #^

1. Cancer Institute, Xuzhou Medical University, Xuzhou, Jiangsu Province, 221004, People’s Republic of China.

2. Center of Clinical Oncology, The Affiliated Hospital of Xuzhou Medical University, Xuzhou, Jiangsu Province, 221004, People’s Republic of China.

3. Jiangsu Center for the Collaboration and Innovation of Cancer Biotherapy, Xuzhou Medical University, Xuzhou, Jiangsu Province, 221004, People’s Republic of China.

4. Nanjing International Hospital Co., Ltd., Nanjing, Jiangsu Province, 210000, People’s Republic of China.

5. Medical Oncology of Huangmei People’s Hospital, Huanggang, Hubei Province, 435500, People’s Republic of China.

6. Department of Oral Medicine, School of Stomatology, Xuzhou Medical University, Xuzhou, Jiangsu Province, 221004, People’s Republic of China.

7. Department of Cancer Biotherapy Center, Third Affiliated Hospital of Kunming Medical University, Kunming, Yunnan Province, 650118, People’s Republic of China.

8. Jiangsu Key Laboratory of Immunity and Metabolism, Department of Pathogenic Biology and Immunology, Xuzhou Medical University, Xuzhou, Jiangsu Province, 221004, People’s Republic of China.

* Xiaoge Gao, Pin Jiang, and Xiaohuan Wei contributed equally to this work.

# Correspondence to:

Prof. Qing Zhang, E-mail: qingzhang@xzhmu.edu.cn; Prof. Xiangye Liu, E-mail: liuxy83@xzhmu.edu.cn; Prof. Hong Yao, E-mail: yaohong20055@hotmail.com.

**Supplementary methods**

**Endotoxin assay**

The endotoxin analysis for purified recombinant proteins (rPK5, rGal-3C, rPK5-RL-Gal-3C) extracted from *E. coli* was performed by using ToxinSensor^TM^ chromogenic LAL endotoxin assay kit (GenScript, Nanjing, China) according to the manufacture’s description. In brief, the standard samples and test samples were serial diluted, respectively. Then, all samples were incubated with LAL, substrate, and stop solutions under endotoxin-free conditions. Finally, the OD value of all samples were read by using microplate reader at 545 nm and the concentration of endotoxin in purified recombinant proteins was calculated according to the standard curve.

**Supplementary data**


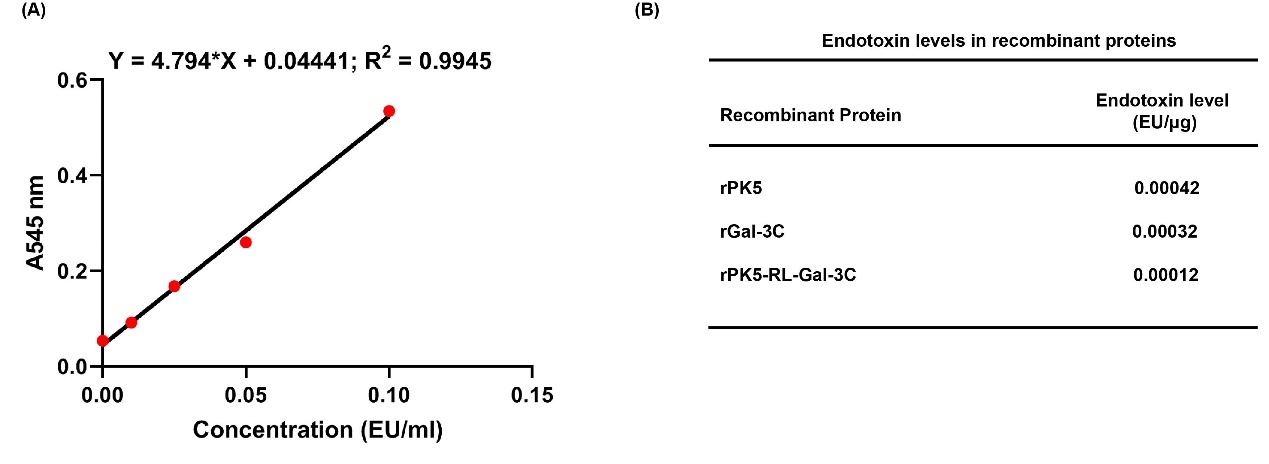


**FIGURE S1. Endotoxin analysis in all purified recombinant proteins.** (A) The standard curve was constructed with R^2^=0.9945. (B) The endotoxin of all purified recombinant proteins was less than 0.0005 EU/μg.


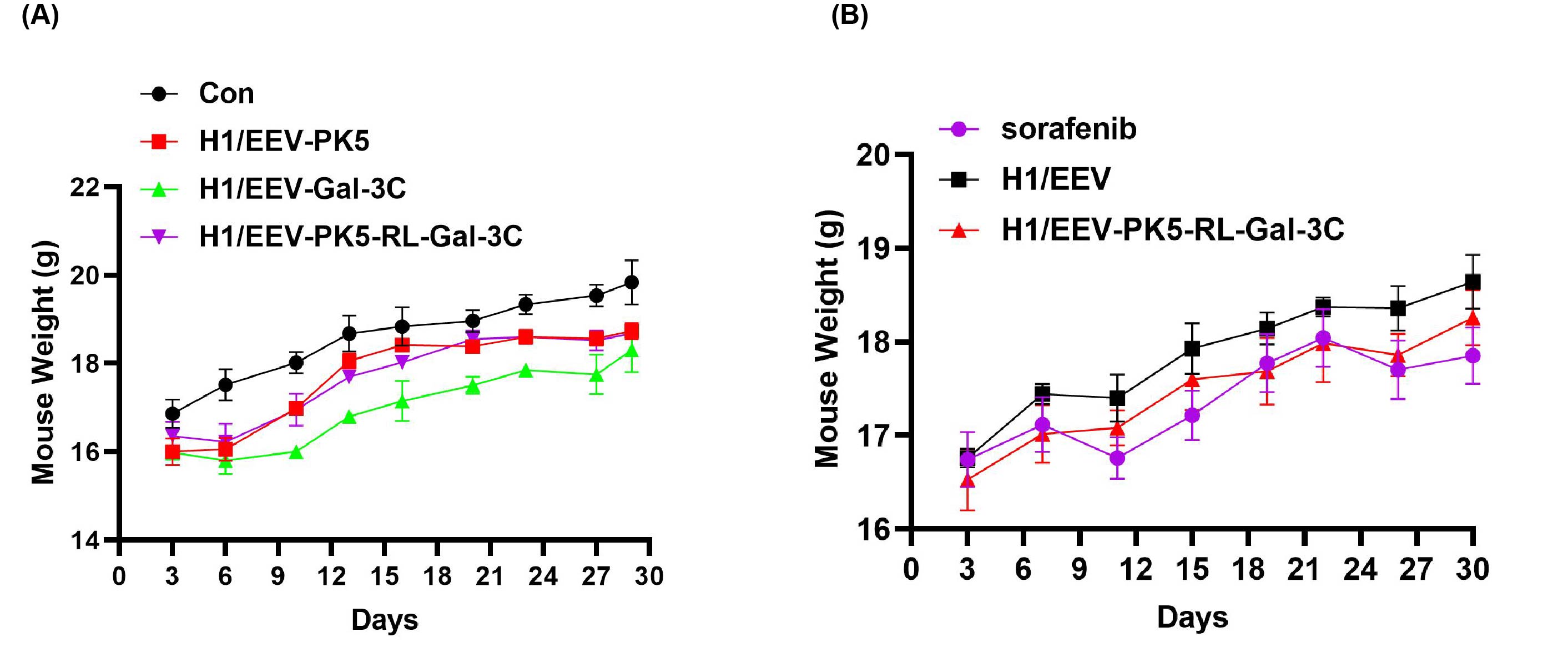


**FIGURE S2. Body weight of tumor-bearing mice.** The body weight of tumor-bearing mice was recorded every three days. No significant differences were presented in the body weight between differential treatment and control tumor-bearing mice. All data shown represent the mean ± SEM.


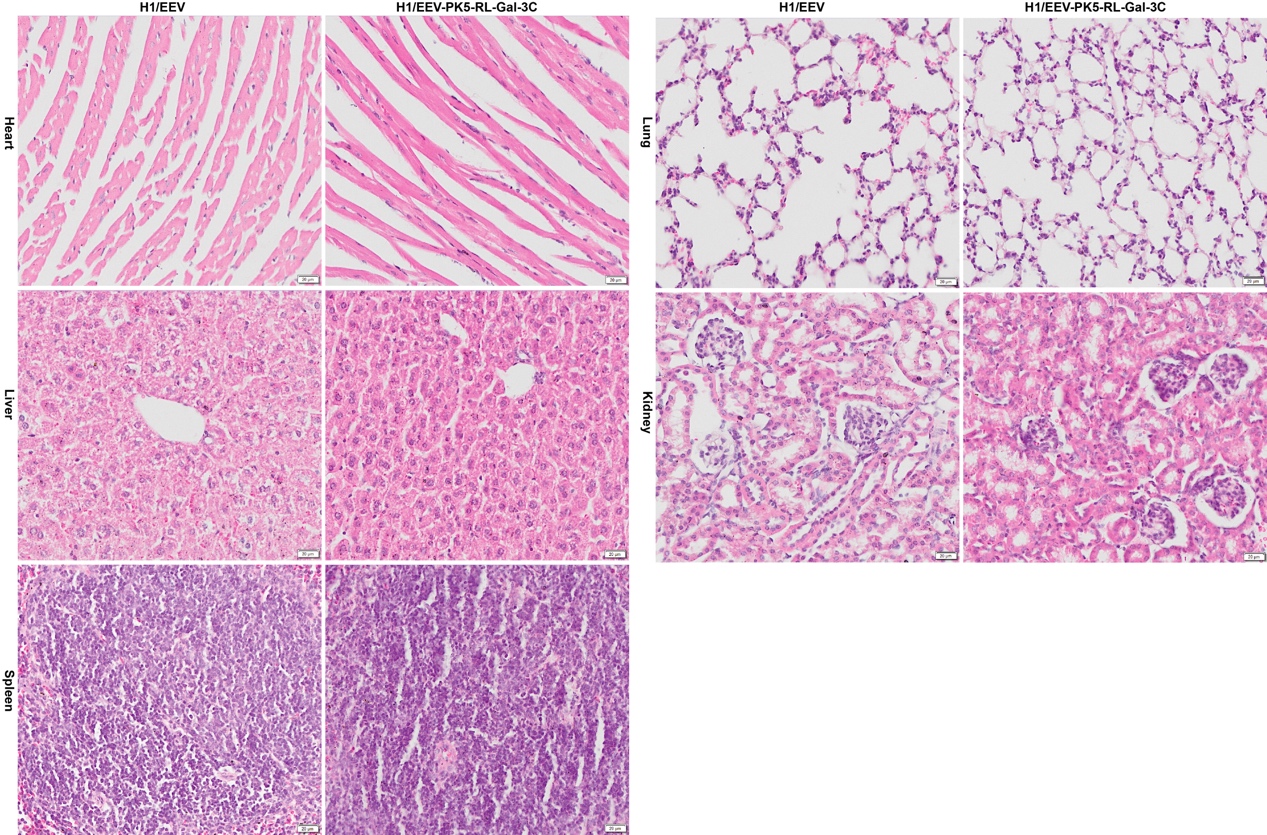


**FIGURE S3. HE staining of internal organs from tumor-bearing mice.** Following treated for 28 days, the internal organs including heart, liver, spleen, lung and kidney from tumor-bearing mice were stained with HE staining. No differences were seen in the organs between differential treatment and control tumor-bearing mice. Scale bar = 20 μm.

**Original gel and western-blotting figures**


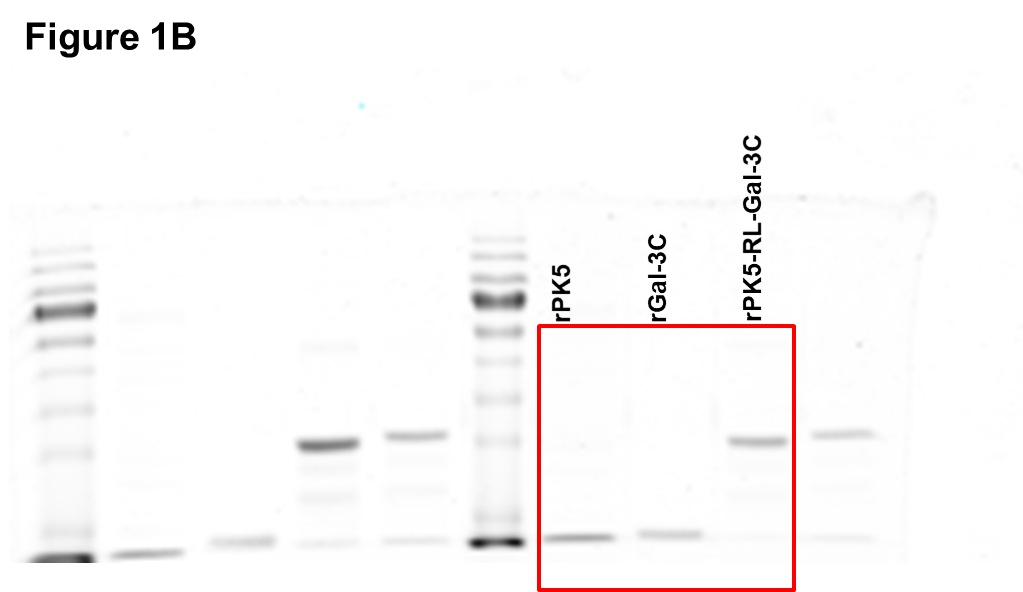


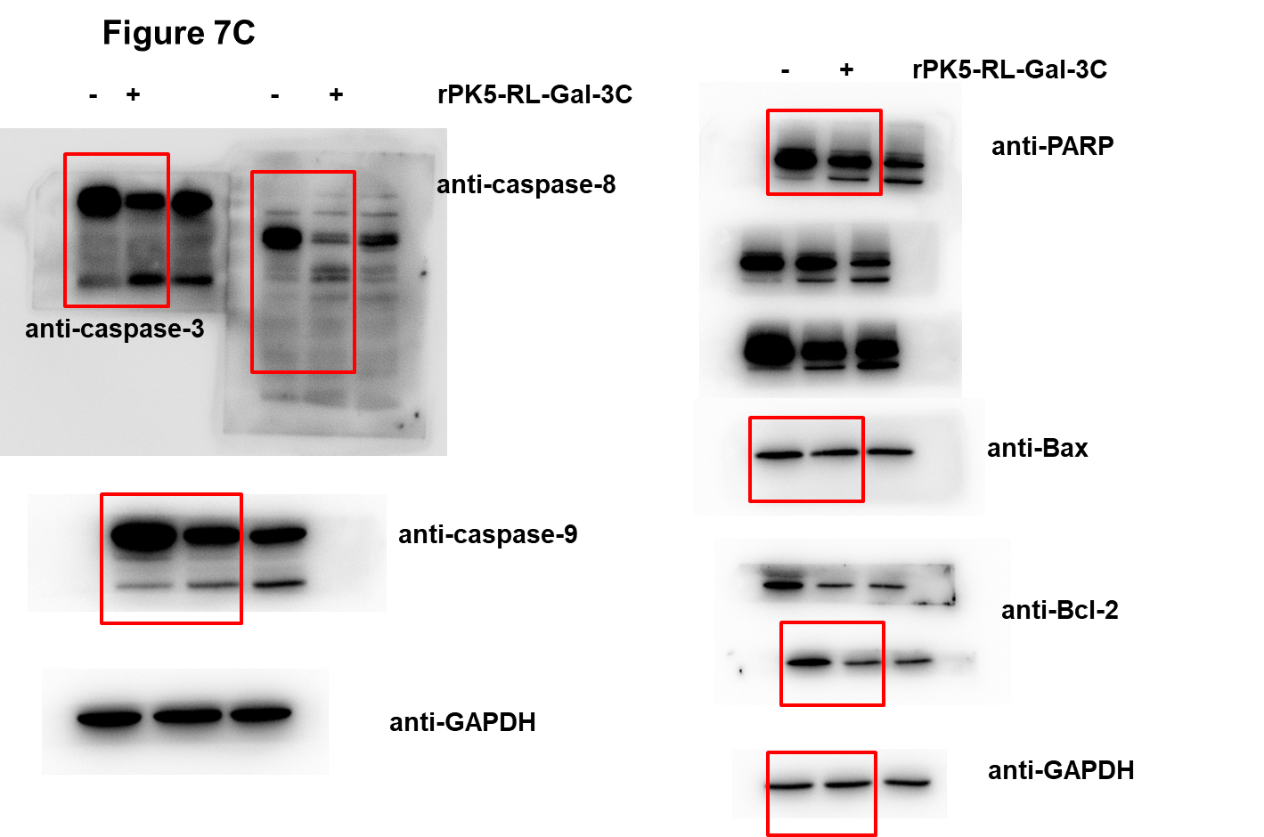

Supplement: Supplementary file 1 — Additional file 1. [file 12885_2023_10608_MOESM1_ESM.doc]
